# Bio-synthesized ZnO nanoparticles and sunlight-driven photocatalysis for environmentally-friendly and sustainable route of synthetic petroleum refinery wastewater treatment

**DOI:** 10.1038/s41598-023-47554-2

**Published:** 2023-11-27

**Authors:** A. El Golli, S. Contreras, C. Dridi

**Affiliations:** 1 Center of Research on Microelectronics and Nanotechnology of Sousse, NANOMISENE Laboratory LR16CRMN01, Technopole of Sousse, B.P. 334, Sousse, Tunisia; 2 Departament d’Enginyeria Química, Universitat Rovira i Virgili, Av. Països Catalans, 26, 43007 Tarragona, Spain; 3 High School of Sciences and Technology of Hammam Sousse, University of Sousse, Sousse, Tunisia

**Keywords:** Materials science, Environmental impact, Physics, Chemical physics, Nanoscience and technology, Nanoscale materials, Other nanotechnology, Chemistry, Biosynthesis, Catalysis, Chemical engineering, Green chemistry, Materials chemistry, Physical chemistry

## Abstract

The design of a green photocatalytic system that harnesses renewable and eco-friendly constituents holds the potential to offer valuable insights into alternative strategies for treating toxic multi-components in refinery water effluents. A significant challenge in implementing a practical and viable approach is the utilization of solar energy—an abundant, natural, and cost-effective resource—for photochemical processes within advanced oxidation processes. In this study, we explored the use of zinc oxide nanoparticles (ZnO NPs) as photocatalyst prepared via an environmentally friendly synthesis approach, resulting in the formation of crystalline wurtzite nanoparticles, with an average size of about 14 nm relatively spherical in shape. Notably, the extract derived from *Moringa oleifera* was employed in this investigation. These nanoparticles were characterized and validated using various characterization techniques, including X-ray diffraction, transmission electron microscopy, field emission scanning electron microscopy, and energy dispersive X-ray spectroscopy. For comparison, conventionally synthesized ZnO NPs were also included in the evaluations. The findings reveal that, under illumination, biosynthesized ZnO nanoparticles (NPs) exhibit photocatalytic performance in effectively breaking down the organic compounds present in synthetic petroleum wastewater. Photochemical analysis further illustrates the degradation efficiency of Green-ZnO, which, within 180 min of irradiation resulted in 51%, 52%, 88%, and 93% of removal for Phenol, O-Cresol. Under optimal loading conditions, NPs produced via the green synthesis approach perform better when compared to chemically synthesized ZnO. This significant improvement in photocatalytic activity underscores the potential of eco-friendly synthesis methods in achieving enhanced water treatment efficiency.

## Introduction

Water is an indispensable valuable resource used in a variety of industrial operations and is essential to all kinds of life. Environmental pollution caused by dangerous chemicals has recently become one of the biggest issues facing industrialized countries. One of the industries that produce a lot of wastewater is the petroleum refining industry. Wastewater produced by the petroleum industries contains a variety of substances, mostly organic molecules (primarily aromatic and aliphatic hydrocarbons) and total solids dissolved (such as salts, barium, and strontium).

Crude oil includes considerable quantities of monoaromatic hydrocarbons including toluene, benzene, ethylbenzene, and xylene (BTEX), which are categorized under volatile organic compounds (VOCs), and trace levels of polycyclic aromatic hydrocarbons (PAHs)^1^. These toxic compounds find their way into the delicate ecological balance via the discharge of wastewater from petroleum industrial facilities, permeating the air, soil, and water, thereby exacerbating environmental pollution^2^. The ecosystem and living species are seriously threatened by a rise in toxins in the water bodies, which can have severe and long-lasting effects Indeed, this can harm aquatic life, disrupt the food chain, and potentially affect human health if contaminated water is used for drinking, irrigation, or other purposes^3^.

Refinery effluents are subject to strict regulations and monitoring to ensure that these potentially harmful compounds are controlled and reduced to safe levels before discharge into the environment. To combat the environmental challenges posed by the release of toxic compounds from petroleum industrial plants, it is essential to promote the adoption of recycling and sustainable practices in the industry. These efforts not only contribute to environmental protection but also offer economic benefits and support long-term water resource preservation^4^.

This urgent concern has prompted a concerted effort to discover renewable technologies for water remediation with the following key tenets: increased efficiency and self-sufficiency. Petroleum refineries commonly employ primary and secondary wastewater treatment techniques. In the primary treatment phase, oil–water separation is achieved through physical methods like sedimentation or dissolved air flotation. To tackle impurities, coagulation with chemicals like aluminum hydroxide or ferric hydroxide is utilized, forming sludge. Nevertheless, these technologies come with inherent drawbacks and constraints. Notably, they generate concentrated sludges necessitating further processing and discharge, which can impose financial constraints due to the substantial initial investments demanded^5^. In lieu of conventional treatment methods, Advanced Oxidation Processes (AOPs) offer a promising alternative for swiftly breaking down contaminants in aquatic environments. These innovative techniques involve the generation of hydroxyl radicals (OH.), among other reactive species, which can effectively interact with organic compounds and facilitate their complete mineralization^6^. Such processes include UV^7^; O_3_/H_2_O_2_^8^; O_3_/UV^7,8^; photo-Fenton and Fenton processes^4,9^ and photo-catalysis^4^.

Among these techniques, solar photocatalysis is attracting considerable interest as a sustainable and environmentally friendly technology for petroleum refineries wastewater treatment owing to its ability to oxidize a wide range of organic pollutants.

Presently, a variety of semiconductor-based nanophotocatalysts have been applied in water pollution remediation, with a significant emphasis on metal oxide nanoparticles^10–12^. Notably, zinc oxide (ZnO) has garnered considerable attention in this regard. ZnO stands out due to its outstanding charge transport properties, characterized by a 3.3 eV bandgap and a high excitation binding energy of 60 meV. Moreover, it exhibits excellent chemical stability, non-toxic nature and long-term photo-stability. These distinctive properties together produce an impressive photocatalytic behavior^13^.

Fundamentally, when a semiconductor boasting a suitably wide band gap absorbs light energy surpassing its own, it prompts the migration of valence band electrons (e^−^) to the conduction band (CB), creating vacancies, or holes (h^+^), in the valence band (VB). These photo-excited electrons and holes subsequently initiate redox reactions with species whose redox potentials align appropriately. This interplay indeed induces reduction and oxidation reactions, giving rise to the production of superoxide (O_2_^.^) and hydroxyl (OH^.^) radicals, which play a pivotal role in breaking down the organic pollutant^14,15^.

Indeed, the generated hydroxyl radicals, known for their strong oxidation properties, will initiate the breakdown of the contaminants adhered to the photocatalyst’s surface, leading to the prompt formation of intermediate substances. These intermediates will ultimately transform into environmentally friendly compounds like carbon dioxide (CO_2_) and water (H_2_O), as indicated in (Eq. 10).

Thus, the process of solar-induced photodegradation of toxic organic substances through redox reactions can be outlined in the following manner^16,17^:

1 $$ {\text{ZnO }} + {\text{h}}\upsilon  \to {\text{ZnO }}({\text{h}}_{{{\text{VB}}}}^{ + } + {\text{e}}_{{\,{\text{CB}}}}^{ - } ) $$

2 $$ {\text{H}}_{{2}} {\text{O }} + {\text{ h}}_{{{\text{VB}}}}^{ + } \to {\text{OH}}^{ \cdot } + {\text{ H}}^{ + } $$

3 $$ {\text{O}}_{{2}} + {\text{e}}_{{{\text{CB}}}}^{ - } \to {\text{O}}_{{2}}^{ - \cdot } $$

4 $$ {\text{O}}_{{2}}^{ - \cdot } + {\text{H}}^{ + } \to {\text{HO}}_{{2}}^{ - } $$

5 $$ {\text{HO}}_{{2}}^{ - } + {\text{ H}}^{ + } \to {\text{H}}_{{2}} {\text{O}}_{{2}} $$

6 $$ {\text{H}}_{{2}} {\text{O}}_{{2}} + {\text{h}}\upsilon \to {\text{2OH}} \cdot $$

7 $$ {\text{Harmful}}\,{\text{compounds}}\,{\text{that}}\,{\text{are}}\,{\text{in}}\,{\text{interaction}}\,{\text{with}}\,{\text{OH}}^{.} \,{\text{Or}}\,{\text{O}}^{{{2} - \cdot }} \to {\text{Intermediates}} \to {\text{harmless}}\,{\text{product}} $$

**Figure 1:**
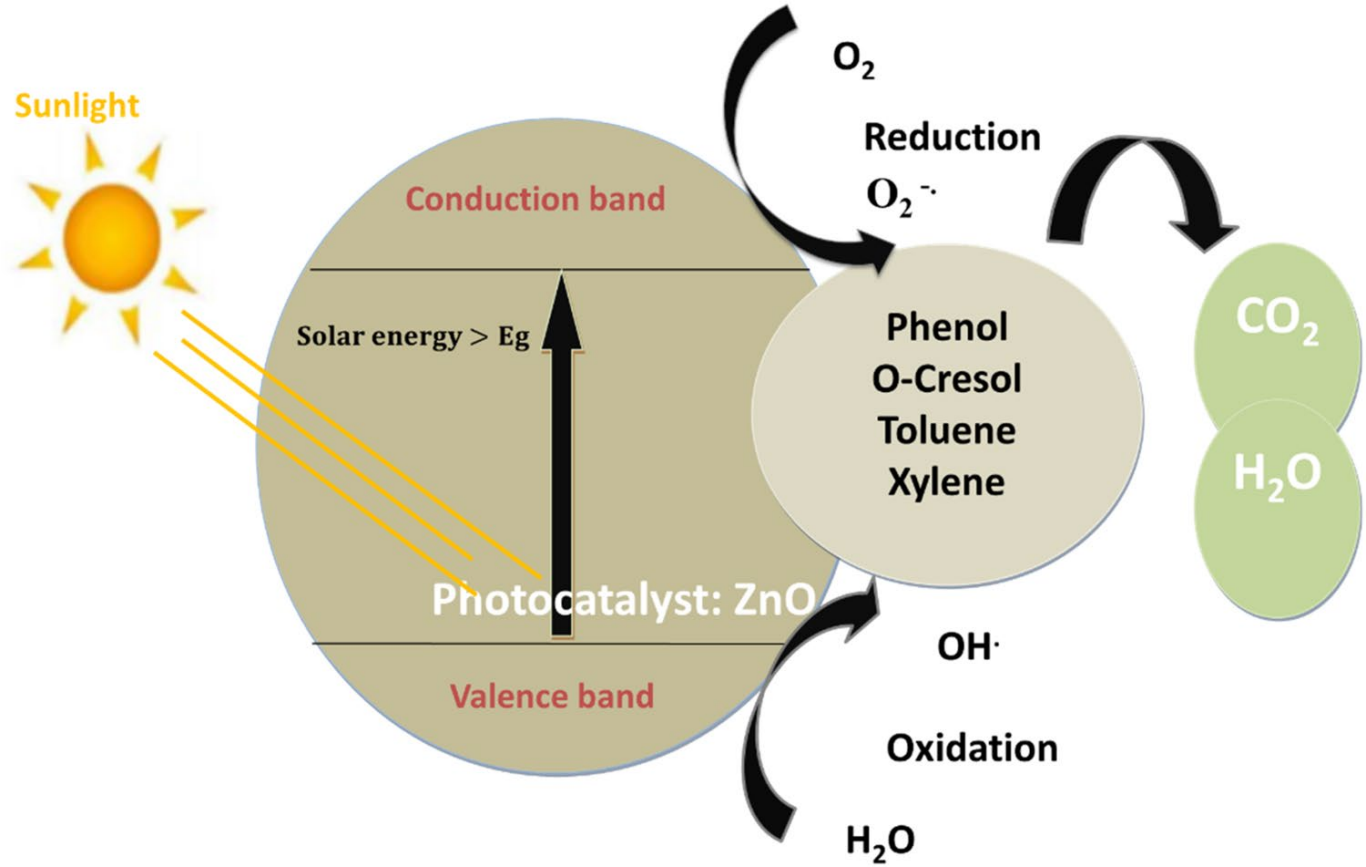
Scheme of PC mechanism occurring over ZnO.

Four aromatic and aliphatic hydrocarbons often discovered in refinery effluent were designated as target pollutants to evaluate their removal effectiveness by solar-assisted photocatalysis, specifically, Phenol, O-Cresol, Toluene, and Xylene. Figure 1 illustrates the redox reaction occurring during photocatalysis over ZnO NPs.

Several methods for NPs synthesis are under consideration. They include both physical and chemical approaches, which, while effective, often entail high costs, time consuming, and environmentally toxic. The environmental compatibility is a significant advantage when it comes to applications involving water treatment, as it ensures that no harmful byproducts or residual toxicity are introduced during the remediation process. Indeed, in conventional processes, common reducing agents, such as sodium citrate, sodium borohydride, and various alcohols are widely known for their hazardous properties, their toxicity, flammability, explosiveness, and resistance to decomposition. Therefore, nowadays different researchers attempted to provide safer, non-toxic, and ecofriendly approaches for NPs fabrication. One such innovative approach is the biological synthesis of NPs. In the process of green synthesis, a natural extract such as microorganisms and/or plant extracts is harnessed as an environmentally sustainable alternative reducing and capping agent. Consequently, the resulting NPs are devoid of any remnants of organic solvents or toxic chemicals, rendering them inherently eco-friendly when introduced into the environment. This approach offers a distinct advantage over conventional methods, due to its simplicity, cost-effectiveness, environmental friendliness, and relative reproducibility^18–22^.

Based on several studies, green synthesis presents an alternative and promising approach to produce NPs that are safer, with reduced chemical toxicity, benefiting both human health and the environment^23–26^. Jayarambabu et al. aimed to synthesize ZnO NPs using Lawsonia inermis leaf extract and explore their potential toxicological impacts. The histopathological assessment revealed the safe use of biosynthesized ZnO NPs, confirming their non-toxicity and compatibility with biological systems, thus indicating their promise in the treatment of various diseases. This study conclusively establishes the harmlessness of the biogenic production of ZnO NPs on all vital organs^27^. Moreover, early literature has shown that, compared to conventionally produced NPs, biosynthesized ZnO NPs significantly suppress both bacterial and fungal diseases^28^. Furthermore, a recent review lends support to the utilization of environmentally-friendly produced ZnO NPs as feed additives, highlighting their potential to enhance immunity against viral infections^24^. ZnO NPs derived from natural resources have received approval from the United States Food and Drug Administration (FDA). They are classified as “GRAS,” which stands for “Generally Recognized As Safe.”^29^. Rashidian et al.^23^ conducted a study to assess the toxicological effects of green synthesized versus commercial ZnO NPs on the immune responses within the skin mucus of carp. The results of this investigation revealed that green ZnO NPs exhibited significantly reduced immunosuppressive effects on important components of fish skin mucus. These green NPs hold immense promise for a wide range of applications in the realms of biology, agriculture, and environmental monitoring. In the future, they have the potential to significantly enhance ecological protection and conservation efforts^30,31^.

This study presents a green synthesis approach for the production of ZnO photocatalysts, designed to support sustainable and environmentally friendly water remediation processes. This method involves the use of safer precursors, the elimination of hazardous compounds, the reduction of energy consumption and the utilization of renewable natural resources.

The added value of our strategy toward water remediation achievements is articulated in 3 aspects:

- Eco-friendly photocatalyst fabrication process, with the addition of few or no chemical compounds during the synthesis, which imposes the respectful aspect to the environment when released into the ecosystem.
- Sustainable and cost-effective nanotechnology. On the one hand it employs a green synthesis using readily accessible and cost-effective plant extracts. On the other hand, it utilizes photocatalysis through natural sunlight instead of artificial lighting, which not only has a shorter operational life but also demands a high energy input. This dual-pronged approach simplifies the process, reduces costs, and enhances scalability for widespread application.
- Simultaneous degradation of organic compounds, present in refinery effluents (Phenol, O-Cresol, Toluene, Xylene).

Therefore, in the present work, we highlight the utilization of *M. Oleifera* leaf, a natural substance as a reducing and capping agent to generate crystalline ZnO NPs. This study illustrates the initial efforts to optimize multicomponent synthetic refinery wastewater’s oxidation processes using solar light, coupled with biosynthesized photocatalyst. The prepared materials were investigated by means of X-ray diffraction (XRD), Transmission electron microscope (TEM), field emission scanning electron microscope (FESEM), and energy-dispersive X-Ray spectroscopy analysis (EDXS). Photocatalytic degradation of synthetic refinery wastewater (SRW) with the prepared catalysts is also reported.

## Methodology of the study

### Biosynthesis of ZnO NPs using Moringa oleifera leaves extract (Green-ZnO)

#### Declaration statement

We declare that the collection of plant material is in accordance with relevant institutional, national and international guidelines and legislation.

#### Preparation of *plant* extract

*Moringa oleifera* leaves have served as an eco-friendly alternative natural reducing and capping agent. The “drumstick tree,” also known as *Moringa oleifera Lam*., is acknowledged as a plentiful and reasonably priced plant. The phytochemical profile of its leaves showed the presence of essential bioactive compounds; vitamins, phenolic acids, flavonoids, and glucosides^32,33^.

*Moringa oleifera* plant has been cultivated and collected from the Higher Agronomic Institute of Chott Mariem (ISA CM), Tunisia. 10 g of cleaned and dried M. Oleifera leaves were boiled for 30 min in 100 mL of double distilled water at 60 °C under magnetic stirrer. The mixture was brought down to room temperature (for 1h45), then filtered through filter paper and a light-yellow solution. The filtered plant extract was kept in a refrigerator at 4 degrees Celsius for future use^34^.

#### Biosynthesis of ZnO NPs

A magnetic stirrer was used to heat 60 mL of M. Oleifera leaf aqueous extract to 80 °C before adding 6 g of zinc nitrate hexahydrate (Zn(NO_3_)_2_.6H_2_O). The mixture was boiled until a yellow-tinted paste formed. After that, it was transferred to a ceramic crucible, then calcined for 2 h in a furnace at 500 °C. Ultimately, a light yellow powder was collected^34^.

### Conventional synthesis of ZnO NPs (Chem-ZnO)

With certain adjustments, the chemical synthesis was created in accordance with the earlier work^35^. After vigorously swirling 6 g of zinc nitrate hexahydrate (Zn(NO_3_)_2_.6H_2_O) into 60 ml of distilled water for 10 min, 2.0 M of ammonium hydroxide was added dropwise until the pH reached 10. The resulting white precipitate was passed through a filter before being calcined for two hours at 500 °C in a furnace.

### Characterization methods of ZnO NPs

Several analytical techniques were used to characterize the ZnO synthesized samples that were shown in the preceding sections. The UV–VIS absorbance spectra were acquired in an integrating sphere using a LAMBDA 365 UV/Vis Spectrophotometer in the range 300–600 nm. An X-Ray Diffractometer was used to characterize the structural properties (Siemens D5000). The angular 2 diffraction ranged from 5 to 70. As a sample holder, a low background Si wafer was employed. A copper X-ray tube provided CuKα radiation (0.15414 nm). The particles morphologies were studied by a FESEM (Thermo Scientific Fisher operated by EDS-Software-pathfinder) associated with EDXS (Oxford INCA PentaFET- × 3). TEM-SAED was used to assess shape, size, and crystallinity. TEM figures were collected using a Transmission Electron Microscope (JEOL model 1011). The solid samples were dispersed in ethanol by sonication, and droplets of zinc oxide NPs suspensions were poured onto a carbon coated-copper grid. Further the material was dried at room temperature and transferred to electron microscope for analysis^36^.

The pH drift technique was used to find the point of zero charge (pHpzc) of the biosynthesized ZnO. A series of 0.01 M NaCl solutions (10 mL each) were formed, and their pH values (pH_0_) were adjusted between 5.0 and 10.0 by adding 0.1 M HCl and 0.1 M NaOH. The suspensions were stirred at 25 °C with 0.02 g of ZnO added to each solution. The solutions’ ultimate pH readings were obtained (pH_f_) after 24 h^37^. The variance between the initial (pH_0_) and final (pH_f_) readings was plotted against the starting pH0 (Y-axis) (X axis). The resultant curve’s intersection generated the pHpzc where pH = 0^38^.

### Photocatalytic experiments

Four aromatic and aliphatic hydrocarbons often discovered in refinery effluent were designated as target pollutants to evaluate their removal effectiveness by solar-assisted photocatalysis. Table 1 shows the composition of the synthetic water based on earlier studies with Real Refinery Wastewaters (RRWs)^7,39–42^. To prepare the SRW, 900 mL of distilled water was first mixed with 5 mg of Triton-X, the necessary salt quantities, and non-soluble compounds. A homogenizer was then used to emulsify the mixture for 30 min. The mixture was then supplemented with the required amounts of soluble organic materials while being vigorously agitated. Prior to use in the tests, the solution was then adjusted into 1000 mL in distilled and agitated for an additional 30 min to guarantee stable wastewater before use in the studies.

**Table 1:** Water characteristics used in the experiments.

Component	Conc (mg/L)
Toluene	10
Nonane	10
Phenol	10
O-Cresol	20
Xylene	10
Ammonium chloride	70
Sodium chloride	247
Sodium bicarbonate	160
SRW TOC	57.53
IC	31.2
pH	8

HPLC was used to determine the concentrations of the contaminants (Phenol, O-Cresol, Toluene, and Xylene) in the synthetic modeling water (Shimadzu LC-20AT). The separation was obtained through a C18 TeknoKroma column (4.6 × 250 mm, 5 micron) and detected at wavelength of 254 nm. The concentrations of each component were evaluated based on their respective calibration curves using standards.

The set of experiments was carried out in a borosilicate reactor with 550 mL of synthetic wastewater as a photoreactor. For 30 min, the liquid was mixed in the dark in order to assure the adsorption of compounds on the solid surface. An air-cooled 1500-W Xenon lamp in a solar box that simulates sunlight and emits light in the 300–800 nm range was used to irradiate the reactor (ATLAS, SUNTEST CPS +). The illumination was adjusted at 250 W/m^2^. The pH of solution was adjusted using dilute sodium hydroxide and hydrochloric acid solutions. The samples collected at fixed time intervals were centrifuged for15 minutes at 8000 rpm in advance of the data analysis.

## Results and discussion

### Characterization of the synthesized NPs

#### UV visible absorption

**Figure 2:**
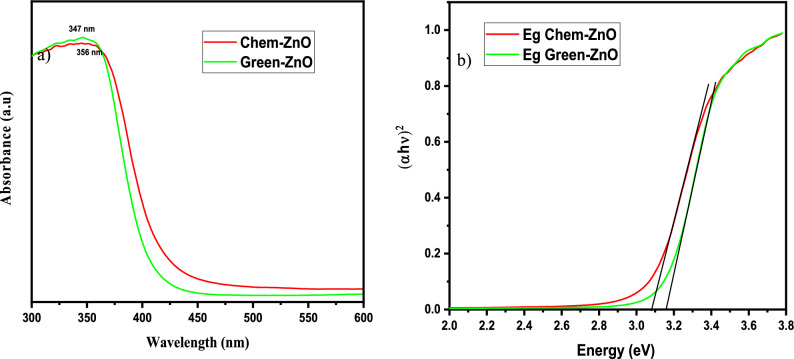
(**a**) UV–Visible absorption spectrum (**b**) Inset. Plot of (αhυ)^2^ versus photon energy of Green-ZnO and Chem-ZnO.

The UV–vis absorption spectra of ZnO materials are depicted in Fig. 2(a). Both samples exhibited UV–vis absorption spectra, with a wide intense absorption from about 350 nm, which may be linked to the intrinsic absorption of the BG of ZnO NPs caused by electron (e−) transfer out from VB towards the CB^34,43–46^.

Since ZnO is a direct band gap Semicond., its ABS coefficient (α) is correlated to the excitation energy by the formula:

$$\mathrm{\alpha h\upsilon }=\mathrm{A}{\left(\mathrm{h\upsilon }-{E}_{g}\right)}^{\mathrm{n}}$$

where A is a proportionality constant, h is the Planck constant, v is the frequency of vibration, and n is an exponent, 1/2, that characterizes direct allowed optical transitions.

E_g_ is calculated by plotting (αhυ)^1/n^ vs. (hυ) and extrapolating to (αhυ)^1/n^ = 0 (Fig. 2b). The extrapolation of the linear part until its intersection with the photon axis was employed to approximate the optical BG. From Fig. 2(b), E_g_ values are 3.16, and 3.07 eV for Green-ZnO, and ZnO-Chem respectively^47–49^. It denotes a widening of the optical BG for the Green-ZnO compared to Chem-ZnO. It is thought that a significant contributing element to this blueshift is the quantum size effect. As the grain size decreases, the continuous energy bands split off into discrete levels, causing the effective expand of the BG. Similar earlier reports also noted these results^44,50,51^.

#### X-ray diffraction (XRD) analysis

**Figure 3:**
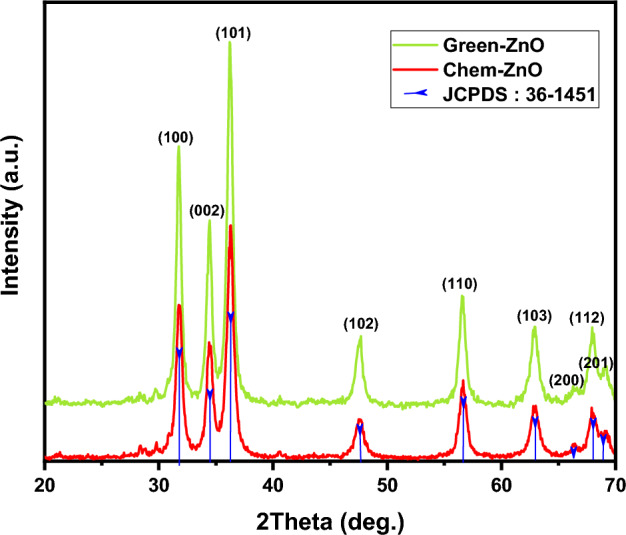
XRD patterns of green and chemically synthesized ZnO NPs.

The XRD patterns of green produced ZnO NPs derived from zinc nitrate hexahydrate and Moringa leaf extract, as well as conventionally generated ZnO NPs formed from zinc nitrate hexahydrate and ammonium hydroxide are illustrated in Fig. 3.

In both cases, the XRD graph demonstrated that the synthesized product was in crystal and that no further impurities could be found once compared to the structure known (ZnO, 04-016-6648). The reference pattern’s hexagonal structure and each of the prominent peaks in the samples were perfectly correlated.

The ZnO NPs' acquired XRD pattern reveals the positions of 2 degree diffraction peaks at 31.75, 34.43, 36.23, 47.55, 56.58, 62.89, 66.42, 68.17, and 69.11 with matching Miller indexes (hkl) of (100), (002), (101), (102), (110), (103), (200), (112), (201) respectively. This demonstrates ZnO’s hexagonal wurtzite phase (JCPDS: 36-1451).

Scherrer’s formula was applied to the high intensity peak (101) to estimate the crystallite size : D = Kλ/β cos θ Where K is a constant (0.9), λ is the X-ray wavelength, and β is full width at half maximum (FWHM)^52^. ZnO NPs have an average crystallite size of 12.51 nm for Green-ZnO and 11 nm for Chem-ZnO. Based on the Debye–Scherrer equation, Both NPs showed almost the same crystallite size. Indeed, in XRD analysis, it should be noted that the crystallite size is assumed to be the size of a coherently diffracting domain and is not necessarily to be the same as the particle size. Furthermore, according to literature, it has been found that the XRD peak can be widened by defects and internal stress^53,54^.

#### FE-SEM analysis

The surface morphology of chemically produced and green ZnO NPs is examined using a FE-SEM.

**Figure 4:**
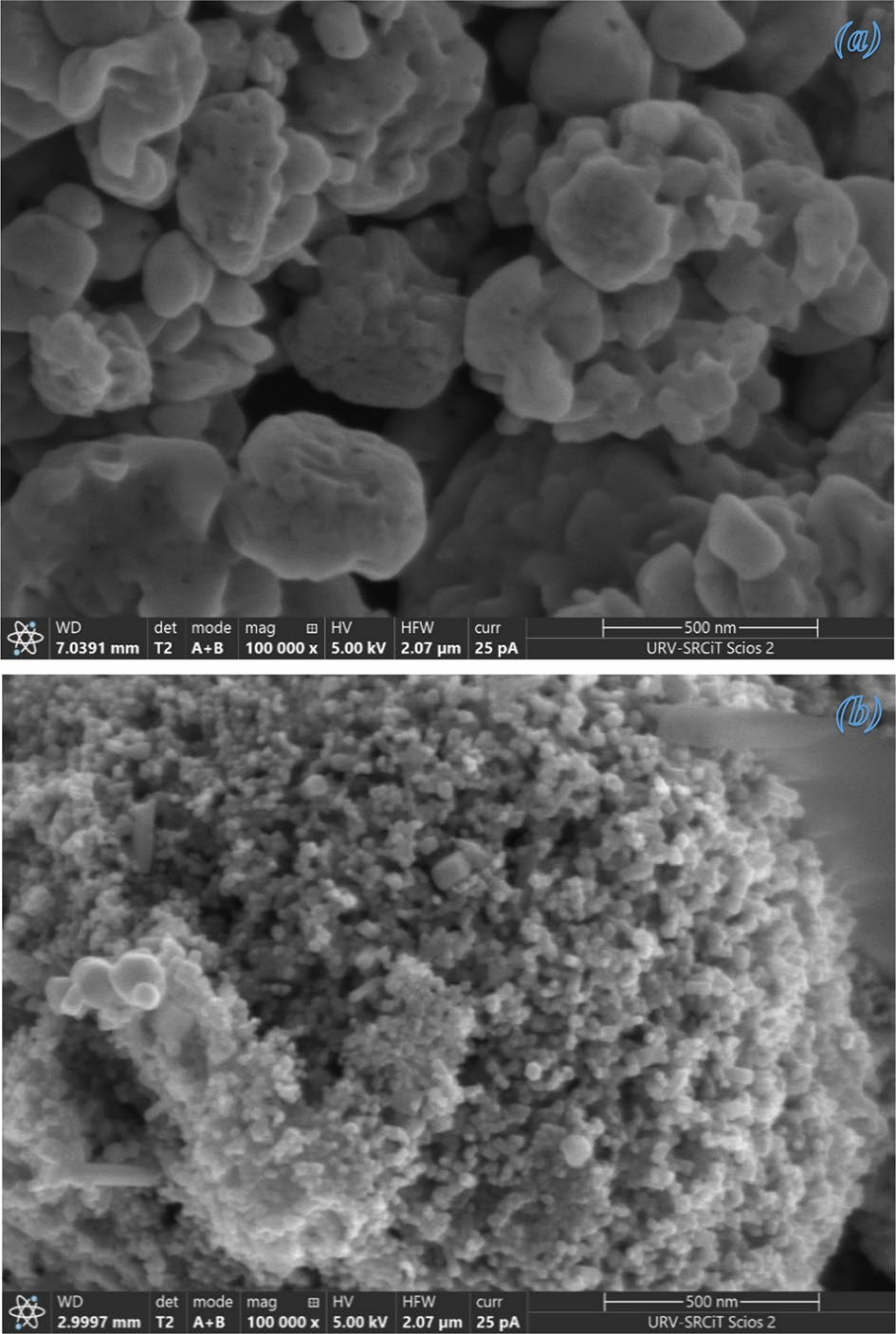
FE-SEM: (**a**) chemically synthesized and (**b**) biosynthesized ZnO NPs.

The SEM image Fig. 4(a) of chemically formed ZnO reveals a range of irregularly shaped NPs clustered. We can observe that the chemically obtained ZnO have no defined geometry. On the other hand, the image of biosynthesized ZnO (Fig. 4b) reveals NPs with well-defined structures at the nanoscale relatively spherical in shape with clear separation^49^. These NPs are surrounded with biomolecules found in the extract, which maintain them apart and avoid agglomeration. This demonstrates that the addition of the plant extract throughout the reaction had a significant influence on the formation mechanism, ending a more defined pattern with less agglomeration^35,55^.

Furthermore, the green method's obtained shape consolidates the physical properties of the NPs, enhancing their qualities and efficiency in many applications. Figure S1 allows us to determine an average size for the biosynthesized ZnO NPs of 13.95 nm. With obvious signals from the atoms of zinc and oxygen and the very low intensity of the carbon atom, the EDX spectrum (Figs. S2 and S3) of produced ZnO NPs reveals their chemical content and validates their purity^49^. The spectrum of Fig. S3 revealed additional peaks corresponding to Magnesium (Mg), Sulfur (S), Chlorine (Cl), Potassium (K) and Calcium (Ca) in very small quantities. Generally, these compounds are contained in the leaf extract of *Moringa oleifera*^55–57^.

#### TEM analysis

**Figure 5:**
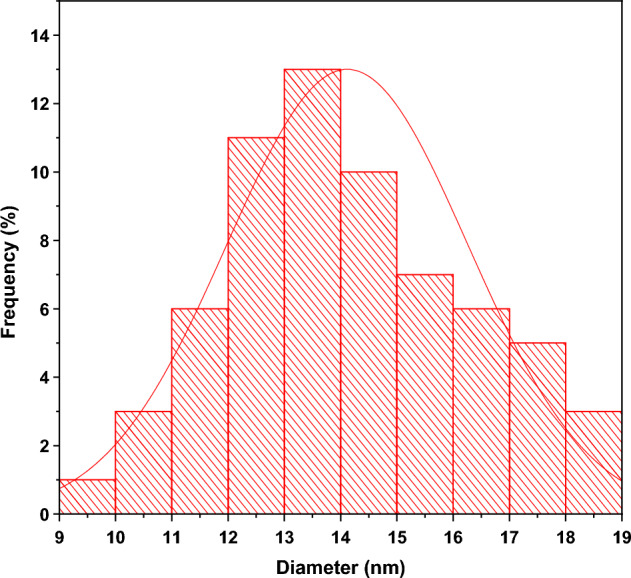
Particle size distribution histogram of Green-ZnO NPs.

TEM images were used to investigate the in-depth properties of chemically synthesized and biosynthesized ZnO NPs. The TEM micrograph of chemically synthesized ZnO in Fig. S4(a) depicts the clustering and irregularity of chemically synthesized ZnO structures. On the other hand, Fig. S4(b) reveals TEM micrograph of the biosynthesized ZnO NPs, giving rise to isolated NPs relatively spherical in shape. This figure (Fig. S4b) is taken at high resolution and confirms the presence of spheroid-like and hexagonal shapes. The histogram in Fig. 5 shows the particle sizes of Green-ZnO NPs ranging from 9 to 18 nm in diameter, with an average size of about 14 nm and a standard deviation of 2.1. These outcomes validate the SEM analysis.

The particles are distributed uniformly which is owing to the existence of organic compounds that encase the particles and act as a capping agent, blocking their aggregation. As a result, it is clear that biosynthesized ZnO has lower particle size and better morphological control than chemically produced ZnO^35^.

The SAED pattern (Fig. S4c–d) was displaying distinct bright dotty rings, demonstrating the particles' crystalline structure, which is consistent with the XRD pattern shown in Fig. 3.The corresponding SAED pattern of the chemically synthesized ZnO displays more discrete spots, indicating the single crystalline nature compared to the biosynthesized, which could be because of the residual organic compounds used during the green fabrication process.

#### Surface area and porosity analyses

Nitrogen adsorption–desorption profiles using BJH and BET techniques were employed to assess the surface properties and the type of porosity of green and chemically produced ZnO NPs. The pore size distribution and porosities were obtained from the desorption isotherm branch by using BJH approach, and the specific surface area was acquired using the BET method.

The specific surface area of the biosynthesized and chemically produced ZnO NPs is 19.789 m^2^/g and 4.923 m^2^/g respectively. The creation of smaller particle sizes may be responsible for the increase in the surface area of green ZnO. Moreover, the pore volumes of Green ZnO and chemically synthesized ZnO are 0.149 cc/g and 0.020 cc/g, respectively. The quantity of ZnO active sites and surface area increase with increasing pore volume^58,59^, so increases the adsorption capacity, which therefore boosts the photocatalytic effectiveness. (Fig. S5a–d).

### Heterogeneous photocatalysis for SRW treatment using ZnO NPs

#### Catalyst loading

Heterogeneous photocatalysis assays were conducted initially at free pH for 180 min with ZnO catalyst loads of 0.1, 0.25, and 0.5 g/L. All studies proceeded with a previous 30-min adsorption stage in the dark. This period of time was determined based on the results of the catalyst’s 60-min adsorption testing. Before the analytical processes, the samples collected were centrifuged for 10 min at 10,000 rpm.

### Adsorption

It can be assumed that 0.1 and 0.25 g/L concentrations show an initial superior adsorption; however, 0.5 g/L concentration exhibits lower adsorption. Considering that the adsorption capacity typically rises with surface area, additional pollutant molecules are adsorbed on the surface given by a high catalyst loading^60^. However, a common tendency of a decline in the removal was seen.

### Photocatalysis

As can be observed, 0.25 and 0.5 g/L achieve nearly identical final results at the end of the 180-min experiment. Analyzing the degradation response reveals that raising the concentration has no discernible effect on photocatalytic performance, which makes 0.25 g/L an optimum catalyst concentration.

#### Effect of initial pH

The surface charge of the semiconductor photocatalyst, the mechanism, and the rate of reactive oxygen species (ROS) formation are all significantly influenced by the solution's pH value^61^. This, in turn, affects the rate of photocatalytic degradation of contaminants^62,63^.

**Figure 6:**
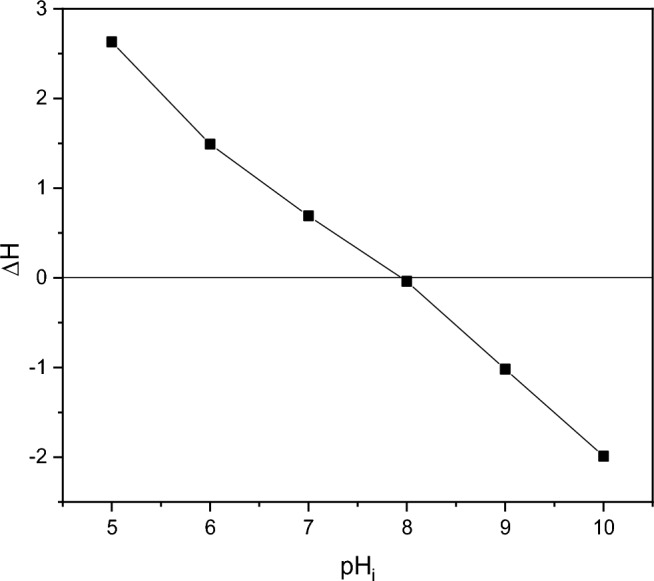
Point of zero charge (pHpzc) of biosynthesized ZnO nanoparticles.

The point of Zero charge pH (pHpzc) of ZnO is 8 according to Fig. 6 and in agreement with previous references^64,65^.

**Figure 7:**
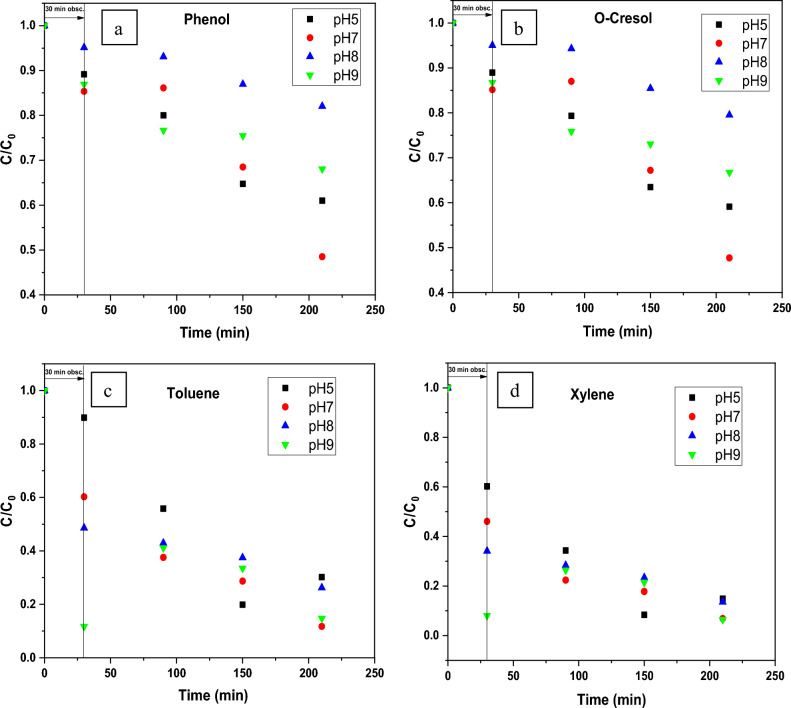
Effect of initial pH on the photocatalytic activity of biosynthesized ZnO photocatalyst toward (**a**) Phenol, (**b**) O-Cresol, (**c**) Toluene, (**d**) Xylene after 30 min dark and 180 min of irradiation.

In this regard, the elimination of each pollutant on ZnO at four distinct pH conditions viz. 5, 7, 8, and 9 have been studied at a constant concentration of catalyst 0.25 g/L. (Fig. 6). The C/Ci vs. time graph (where C denotes the concentration at various time intervals and Ci denotes the compound’s starting concentration) using the biosynthesized ZnO NPs (Green-ZnO) under simulated solar irradiation is shown in Fig. 7(a–d).

The percentage of phenol destroyed is found to be very low in an acidic media. These observations can be related to the phenomenon having positively charged NPs surfaces, which causes protonation of active sites and hence alters phenol adsorption, thereby affecting its removal^66^. Adsorption on ZnO will be less in the basic pH range, where phenol is predicted to be in the ionized state. As a result, surface-mediated degradation will be reduced^67^. Therefore, the neutral pH was the best suited for the phenol degradation.

Giving the basic nature of o-cresol (pKa = 10.316), under acid media, it tends to be positively charged^14,68^. As it is shown, the photodegradation % rose marginally as the pH climbed from 5 to 7. Nevertheless, above the optimum pH 7, there was a decrement in photodegradation%.

The maximum degradation efficiency of Toluene and Xylene (88.30% and 93.13% respectively) was reached with pH 7 following 180 min in the simulated sunlight/ZnO system. Remarkably, the results were comparable at pH values 7 and 9.

The increased removal effectiveness at basic pH can indeed be attributed to the fact that, in alkaline pH, OH^.^ are more readily formed via oxidation of more hydroxyl radicals, that are present on the ZnO surface, thereby boosting the process performance^69,70^.

In all the 4 compounds, at pH8, which is the pH_pzc_ the results are the worse ones. This behavior could be related to a possible aggregation of catalyst particles. Indeed, the zero surface charge creates zero electrostatic surface potential for pH levels near to pH_zpc_, which cannot generate the interaction rejection required to isolate the particles inside the solution. Aggregation occurs as a result, and photocatalyst clusters get bigger^71^.

#### Green vs chemically synthesized ZnO photocatalyst

**Figure 8:**
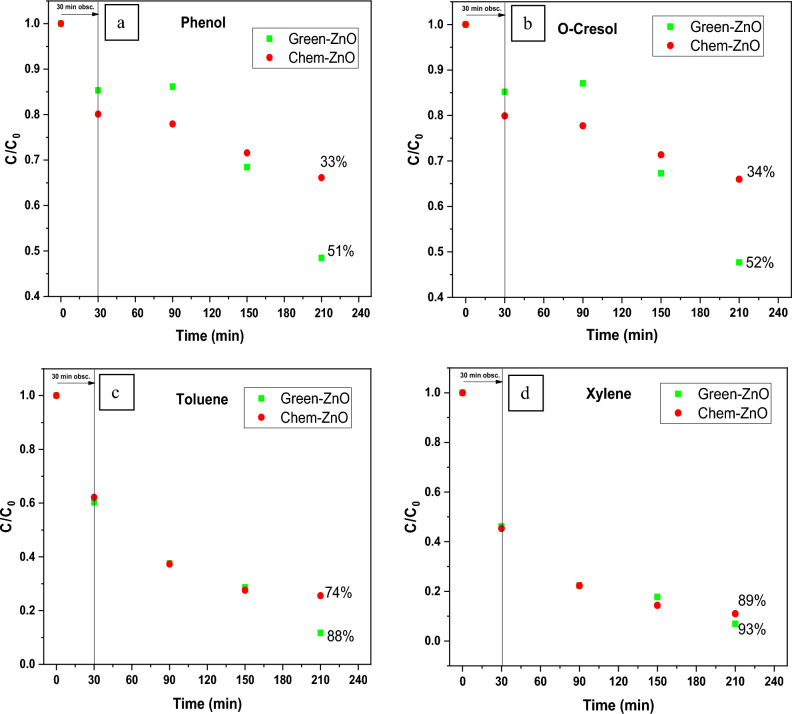
Photocatalytic activity of biosynthesized and chemically synthesized ZnO at optimum conditions toward (**a**) Phenol, (**b**) O-Cresol, (**c**) Toluene, (**d**) Xylene after 30 min dark and 180 min of irradiation.

Figure 8(a–d) illustrates a comparison of time-dependent photocatalytic activity of both the Green-ZnO and the Chem-ZnO NPs towards each pollutant degradation. The photo-chemical analysis revealed that the degradation process with Green-ZnO within 180 min of irradiation resulted in 51%, 52%, 88%, and 93% of removal for Phenol, O-Cresol, Toluene, and Xylene respectively. However, in the case of Chem-ZnO, the percentage of degradation was 33%, 34%, 74%, and 89% of removal for Phenol, O-Cresol, Toluene, and Xylene respectively. The biosynthesis of ZnO clearly demonstrated a higher or quite equivalent degradation effectiveness in comparison to ZnO synthesized with conventional chemical route.

A similar trend has been documented in prior studies that investigated dye degradation, whether using environmentally friendly or chemical synthesis methods^72,73^. It is widely assumed that the morphology, surface, and crystallinity of material are mainly responsible for its photocatalytic activity^74^. Indeed, the plant extract's bioactive substances aid in the development of ZnO nuclei through capping but also stabilizing them. As a result, the green method produces NPs that have greater distribution, structure-tunable, and are size-controlled^75–77^ compared to the chemically synthesized sample, which could provide stability, a larger specific surface area, and reduced particle sizes, thus, high photogenerated charge carrier separation capabilities, enhanced light absorption and finally better degradation of pollutant molecules.

Tables S1, S2, and S3 in the Supplementary Information section offer a comparative analysis of our research findings with those from previous studies focusing on ZnO synthesized through various methods for the PC degradation of petroleum hydrocarbon contaminants. This comparison underscores the competitiveness of the results we have obtained in this study when compared to existing efficiency standards.

The primary objective of this research is to explore an environmentally sustainable, uncomplicated, and cost-effective solution that can either reduce the volume of waste discharged in effluents or promote the reuse of purified water, thus reducing the consumption of freshwater. The novel approach for photocatalyst synthesis employed in this study represents an initial step towards optimizing a more sustainable process, especially, when paired with sun energy, this allows an economically feasible route in the application of solar photocatalysis. Importantly, According to a previous study, the economical evaluation points out that the highest loads for the cost composition are due to catalyst synthesis, corresponding to 95% in a solar photocatalysis system^34^.

Ultimately, working with photocatalysts in form of powder needs a post-processing removal of the NPs from the liquid solution which could be an inefficient additional process step especially from an industrial perspective. For this reason, the development of photocatalysts immobilized as coatings is thus an improvement. That will require modifying the substrate, using a different thickener, or introducing a linker between ZnO and the substrate. In this case, the stability parameter and a study of reusability would be significant. We consider that an interesting topic for our ongoing and future research.

## Conclusion

We reported on the photocatalytic activity of ZnO nanoparticles biosynthesized through a sustainable, cost-effective, easily scalable, and eco-friendly approach. *Moringa oleifera* leaves extract was used as reducing and stabilizer agent, hence playing a significant role towards structural evolution. The reported results reveal that Green-ZnO can be fruitfully exploited for the removal of toxic compounds present in refinery effluents particularly; Phenol, O-Cresol, Toluene, and Xylene with 51%, 52%, 88%, and 93% in sequence. Indeed, the PC efficiency of green-synthesized ZnO NPs is almost equivalent to that of ZnO via a conventional chemical synthesis. The ability of the proposed approach to use sunlight as the only energy input and photocatalysts with low cost and minimal environmental impact underline its significance in the ongoing efforts towards wastewater remediation.

### Supplementary Information

Supplementary Information.

## Data Availability

Data are available from the corresponding author Prof. Chérif Dridi, upon reasonable request.
