# Supplementary material for: Bio-synthesized ZnO nanoparticles and sunlight-driven photocatalysis for environmentally-friendly and sustainable route of synthetic petroleum refinery wastewater treatment
Source: Sci Rep. 2023 Nov 27;13:20809. doi: 10.1038/s41598-023-47554-2 (PMC10682493; doi:10.1038/s41598-023-47554-2)
Supplement: Supplementary file 1 — Supplementary Information. [file 41598_2023_47554_MOESM1_ESM.docx]

**Supplementary Material**

**for**

**Bio-synthesized ZnO nanoparticles and sunlight-driven photocatalysis for Environmentally-friendly and sustainable route of Synthetic Petroleum Refinery Wastewater treatment**

A. El Golli ^1,3^, S. Contreras^2*^, C. Dridi ^1*^

^1^NANOMISENE Laboratory LR16CRMN01, Center of Research on Microelectronics and Nanotechnology of Sousse, Technopole of Sousse, B.P. 334, Tunisia

^2^Departament d’Enginyeria Química, Universitat Rovira i Virgili, Av. Països Catalans, 26, Tarragona, 43007, Spain

*^3^* University of Sousse, High School of Sciences and Technology of Hammam Sousse, Tunisia

* Corresponding authors: sandra.contreras@urv.cat / cherif.dridi@crmn.rnrt.tn


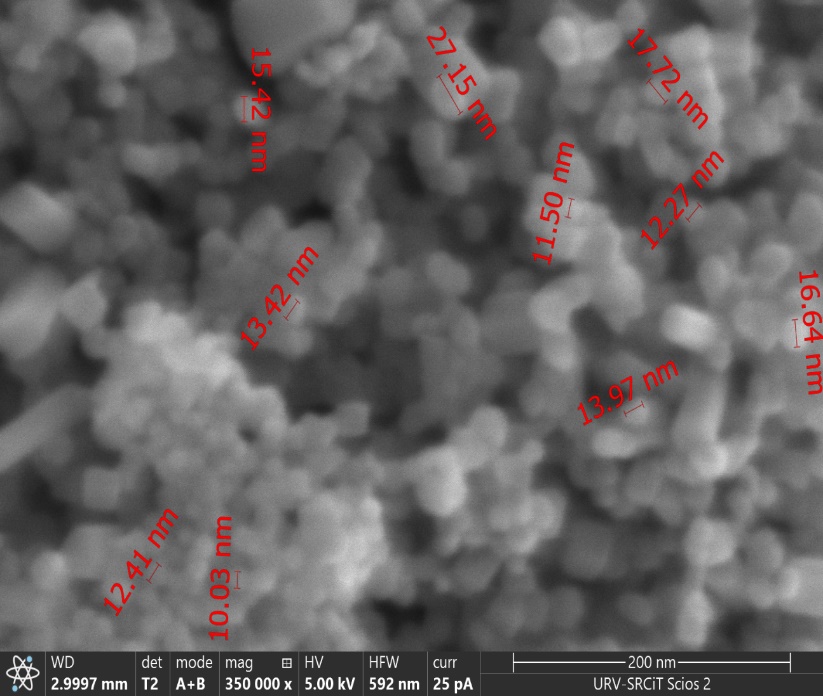

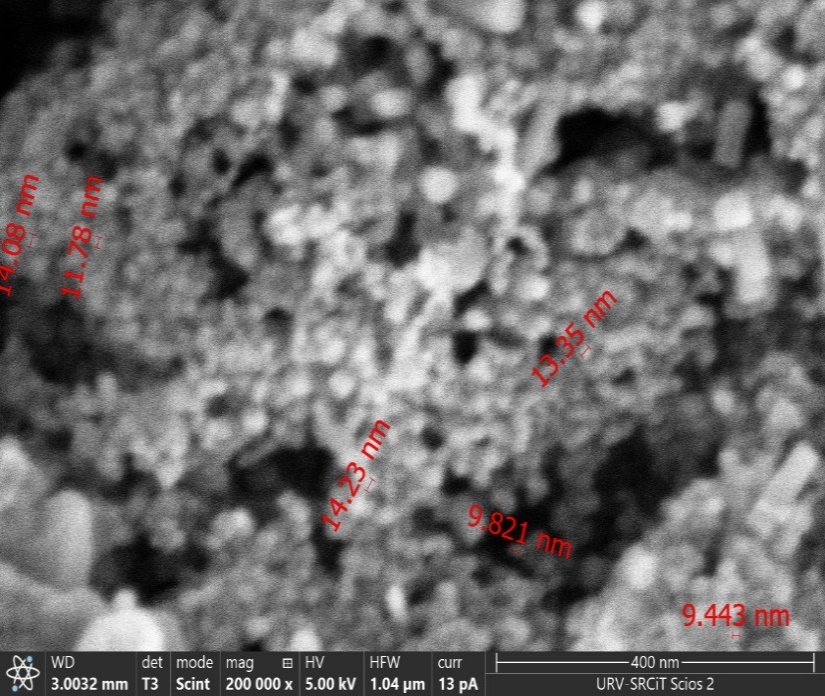


**Fig. S1.** FE-SEM: Size of the biosynthesized ZnO NPs.

***
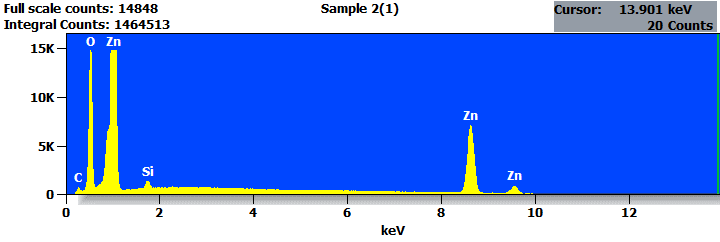
***

******

**Fig. S2.** EDX: Chemically synthesized Zinc Oxide NPs.

***
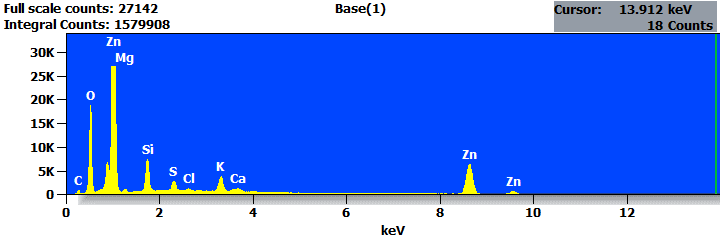
***

******

**Fig. S3.** EDX: Biosynthesized Zinc Oxide NPs.







***(a)***

***(b)***







***(d)***

***(c)***

**Fig. S4.** TEM: ZnO NPs (a) chemically synthesized, (b) biosynthesized, and SAED: ZnO NPs (c) chemically synthesized, (d) biosynthesized.


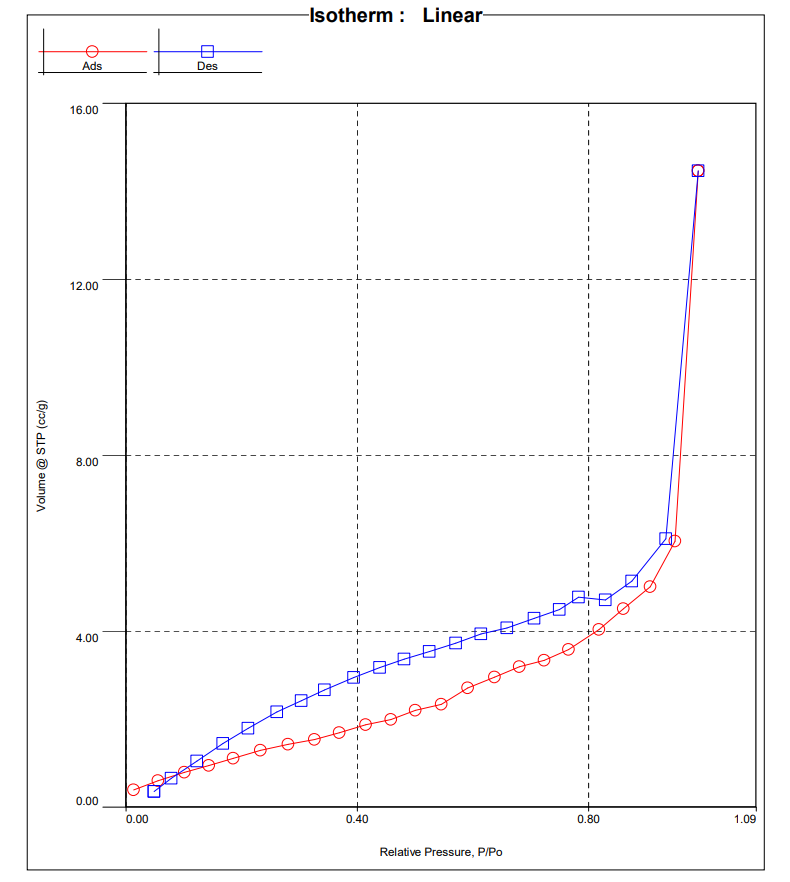

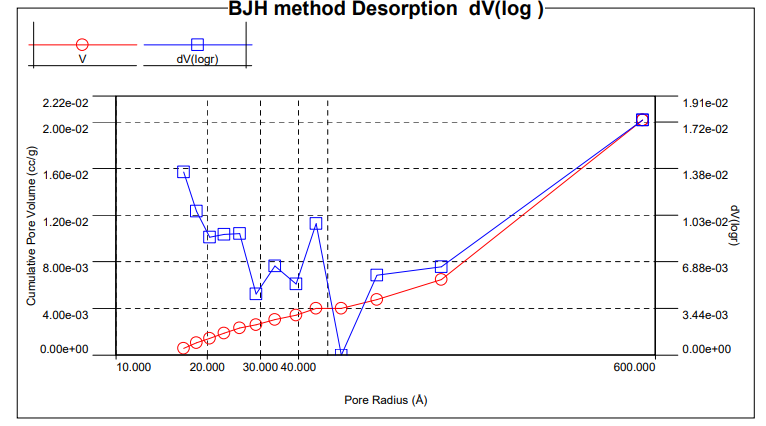

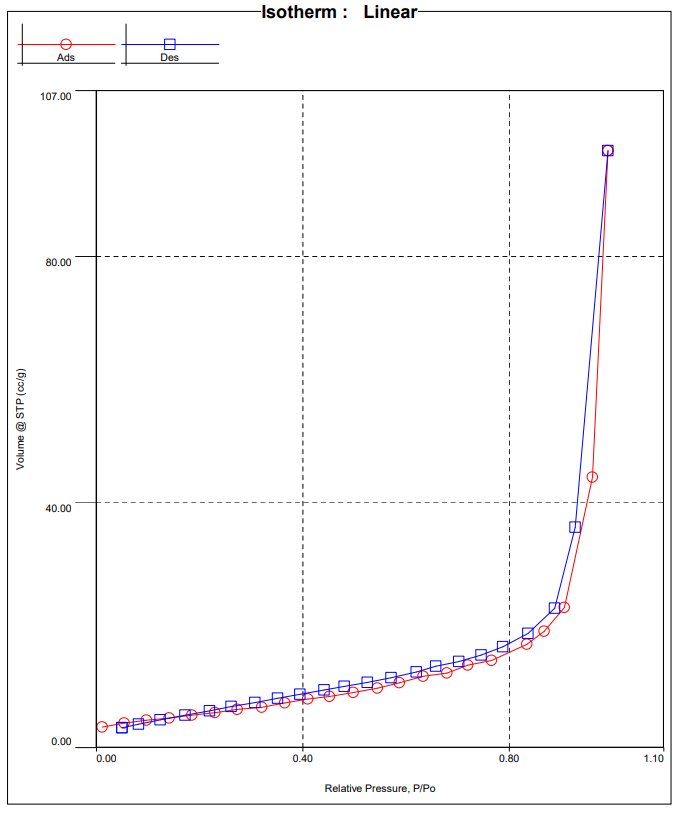

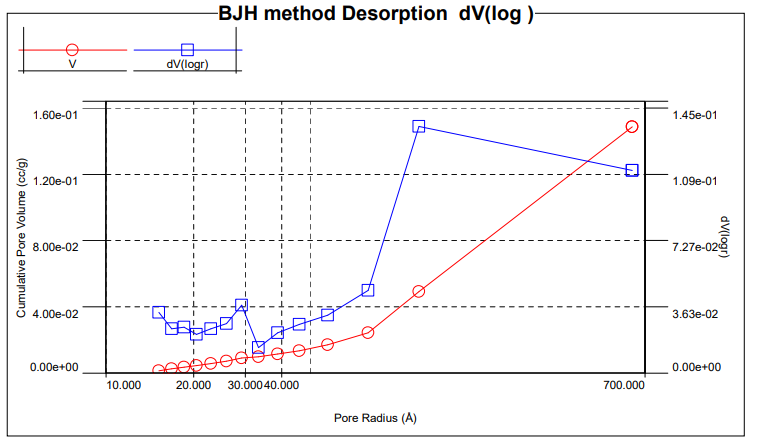


b

a

c

d

**Fig.S5.** Nitrogen adsorption-desorption isotherms and corresponding pore size distribution curves. (a-b) Green-ZnO and (c-d) Chem-ZnO.

**Table S1:** Comparison of photocatalytic performance toward phenol with previously reported literature.

| The process | Synthesis method | Catalyst load  (g/L) | Pollutant conc. (ppm) | Degradation (%) | Ref. |
| --- | --- | --- | --- | --- | --- |
| TiO2/ZnO/H2O2/Vis | Commercial | ZnO (0.59) TiO2 (0.7) | 243 | 37 % in 175 min | ^1^ |
| ZnO/persulphate/ Sunlight | Commercial | 0.4 | 25 | 70 % in 150 min | ^2^ |
| BiOI/ZnO/Sunlight | Hydrothermal | 1 | 25 | 99 % in 120 min | ^3^ |
| Core-shell ZnO/O-g-C_3_N_4_/Vis | Hydrothermal | 2 | 12.3 | 85 % in 120 min | ^4^ |
| ZnO/UV | Hydrolysis & precipitation | 1 | 10 | 56 % in 240 min | ^5^ |
| ZnO nanorods/Visible | Microwave-assisted hydrothermal | - | 10 | 50 % in 150 min | ^6^ |
| ZnO NRs substrates / UV light | hydrothermal | - | 5 | ̴90 % in 600 min | ^7^ |
| Ni@ZnO0.6S0.4/Visible | Precipitation | 0.5 | 320 | 88 % in 180 min | ^8^ |
| Urchin-like ZnO microrod-rGO/Visible | Hydrothermal method | 0.2 | 20 | 84 % in 150 min | ^9^ |
| ZnO NPs/Visible | Green synthesis | 0.15 | - | 70% in 120 min | ^10^ |
| ZnO/Sunlight | Green synthesis | 0.25 | 10 | 51 % at 180 min | This work |

**Table S2:** Comparison of photocatalytic performance toward Cresol with previously reported literature.

| The process | Synthesis method | Catalyst load (g/L) | Pollutant conc. (ppm) | Degradation (%) | Ref. |
| --- | --- | --- | --- | --- | --- |
| ZnO/Visible | Commercial | 1.5 | 25 | 85% in 360 min | ^11^ |
| Mn-doped ZnO/Visible | Precipitation method | 1.5 | 35 | 70% in 360 min | ^12^ |
| ZnO/UV | Commercial | 2.5 | 100 | 93% in 360 min | ^13^ |
| ZnO | Commercial | - | 100 | 43% in 360 min | ^14^ |
| ZnO/Sunlight | Green synthesis | 0.25 | 10 | 52% 180 min | This work |

**Table S3:** Comparison of photocatalytic performance toward Toluene and Xylene with previously reported literature.

| The process | Synthesis method | Catalyst load (g/L) | Pollutant conc. (ppm) | Degradation (%) | Ref. |
| --- | --- | --- | --- | --- | --- |
| V 2 O 5 /zinc – ferrite/Visible | Solvothermal | 0.5 | 50 | 95% in 90 min | ^15^ |
| ZnO Nanoparticles / UV | - | 80 gr/m 2 | 600 | 76% 300 min | ^16^ |
| ZnO nanorods/Visible | Microwave assisted hydrothermal | - | 10 | 90% in 180 min | ^17^ |
| ZnO-graphene (GZ-15%)/UV | Microwave synthesis | 0.1 | 40 | 70% in 45 min | ^18^ |
| Fe-ZnO/SA (20%)/Visble | Sol-gel | 1 | - | 82% (Tol) and 40 % (Xyl) in 120 min | ^19^ |
| ZnO/Sunlight | Green synthesis | 0.25 | 10 | 88 % (Tol) and 93 % (Xyl) in 180 min | This work |

**References**

1. Aljuboury, D. A. D. A. & Shaik, F. Assessment of TiO2/ZnO/H2O2 Photocatalyst to treat wastewater from oil refinery within visible light circumstances. *South African Journal of Chemical Engineering* **35**, 69–77 (2021).

2. Shukla, P. R., Wang, S., Ang, H. M. & Tadé, M. O. Photocatalytic oxidation of phenolic compounds using zinc oxide and sulphate radicals under artificial solar light. *Separation and Purification Technology* **70**, 338–344 (2010).

3. Jiang, J. *et al.* Enhanced photocatalytic degradation of phenol and photogenerated charges transfer property over BiOI-loaded ZnO composites. *Journal of Colloid and Interface Science* **494**, 130–138 (2017).

4. Xing, Z. *et al.* Synthesis of core-shell ZnO/oxygen doped g-C3N4 visible light driven photocatalyst via hydrothermal method. *Journal of Alloys and Compounds* **708**, 853–861 (2017).

5. Ye, J., Li, X., Hong, J., Chen, J. & Fan, Q. Photocatalytic degradation of phenol over ZnO nanosheets immobilized on montmorillonite. *Materials Science in Semiconductor Processing* **39**, 17–22 (2015).

6. Al-Sabahi, J., Bora, T., Al-Abri, M. & Dutta, J. Controlled Defects of Zinc Oxide Nanorods for Efficient Visible Light Photocatalytic Degradation of Phenol. *Materials* **9**, 238 (2016).

7. Daher, E. & The Society of Digital Information and Wireless Communication. Photocatalytic degradation of phenolic effluents in petroleum refineries. *IJEETDM* **5**, 22–29 (2019).

8. Dahash, M. Sh., Ammar, S. H. & Abdulnabi, W. A. Synthesis of magnetic zincoxysulfide core/shell nanocomposites (Ni@ZnO _0.6_ S _0.4_ ) for COD photocatalytic degradation in oil refinery wastewater. *IOP Conf. Ser.: Mater. Sci. Eng.* **928**, 022063 (2020).

9. Margaret, S. M., Paul Winston, A. J. P., Muthupandi, S., Shobha, P. & Sagayaraj, P. Enhanced Photocatalytic Degradation of Phenol Using Urchin-Like ZnO Microrod-Reduced Graphene Oxide Composite under Visible-Light Irradiation. *Journal of Nanomaterials* **2021**, 1–11 (2021).

10. Sharmila, M. *et al.* Photocatalytic and Biological Activity of ZnO Nanoparticles Using Honey. *Coatings* **11**, 1046 (2021).

11. Abdollahi, Y., Abdullah, A. H., Zainal, Z. & Yusof, N. A. Photodegradation of o-cresol by ZnO under UV irradiation. *Vol No.*

12. Abdollahi, Y., Abdullah, A. H., Gaya, U. I., Zainal, Z. & Yusof, N. A. Enhanced photodegradation of *o* -cresol in aqueous Mn(1%)-doped ZnO suspensions. *Environmental Technology* **33**, 1183–1189 (2012).

13. Abdollahi, Y., Abdullah, A. H., Zainal, Z. & Yusof, N. A. Photocatalytic Degradation of p-Cresol by Zinc Oxide under UV Irradiation. *IJMS* **13**, 302–315 (2011).

14. Brooms, T. J., Otieno, B., Onyango, M. S. & Ochieng, A. Photocatalytic degradation of P-Cresol using TiO _2_ /ZnO hybrid surface capped with polyaniline. *Journal of Environmental Science and Health, Part A* **53**, 99–107 (2018).

15. Mohan, H. *et al.* Enhanced visible light photocatalysis with E‐waste‐based V _2_ O _5_ / zinc–ferrite : BTEX degradation and mechanism. *J of Chemical Tech & Biotech* **95**, 2842–2852 (2020).

16. Keramati, M. & Ayati, B. Petroleum wastewater treatment using a combination of electrocoagulation and photocatalytic process with immobilized ZnO nanoparticles on concrete surface. *Process Safety and Environmental Protection* **126**, 356–365 (2019).

17. Al-Sabahi, J., Bora, T., Al-Abri, M. & Dutta, J. Efficient visible light photocatalysis of benzene, toluene, ethylbenzene and xylene (BTEX) in aqueous solutions using supported zinc oxide nanorods. *PLoS ONE* **12**, e0189276 (2017).

18. Ahmed, G. *et al.* ZnO flowers and graphene oxide hybridization for efficient photocatalytic degradation of o-xylene in water. *Materials Chemistry and Physics* **212**, 479–489 (2018).

19. Najafidoust, A. *et al.* Sequential impregnation and sol-gel synthesis of Fe-ZnO over hydrophobic silica aerogel as a floating photocatalyst with highly enhanced photodecomposition of BTX compounds from water. *Solar Energy* **225**, 344–356 (2021).
